# Revealing the Mechanism of *In Vitro* Wound Healing Properties of *Citrus tamurana* Extract

**DOI:** 10.1155/2013/963457

**Published:** 2013-05-02

**Authors:** Madhyastha Harishkumar, Yamaguchi Masatoshi, Sameshima Hiroshi, Ikenoue Tsuyomu, Maruyama Masugi

**Affiliations:** ^1^Department of Applied Physiology, Faculty of Medicine, University of Miyazaki, Miyazaki 889-1692, Japan; ^2^Department of Obstetrics and Gynecology, Faculty of Medicine, University of Miyazaki, Miyazaki 889-1692, Japan

## Abstract

In the present investigation, we examined the effect of Hyuganatsu (*Citrus tamurana*) extract (HE) on skin fibroblast (TIG-119) proliferation and migration during *in vitro* wound healing. HE selectively inhibited proliferation of TIG-119 cells at higher concentration (>1.0 mg/mL); at lower concentrations (0.1, 0.25, 0.5, and 0.75 mg/mL), it exhibited linear and time-dependent cell proliferation. *In vitro* scratch wound healing studies showed that the HE also accelerated the migration of cells towards the wounded region. Cytometric analysis demonstrated that HE extract did not alter G_1_/0 and S phases of cell cycle in any concentration studied; however, G_2_/M phases of cell cycle were significantly (*P* < 0.05) accelerated at 0.75 mg/mL dose. RT-PCR and Western blotting analysis indicated that HE markedly overexpressed levels of Rac-1, Rho-A, and Cdc-42 mRNA and the respective proteins. Cyclin-dependent kinases (Cdk-1 and -2) gene expression activity was significantly (*P* < 0.05) increased, but protein content decreased during treatment with HE. The induction of Cdk-1 and -2 by HE was abolished by inhibitors, transcription (DRB), and translation (CHX), implying transcriptional regulation that required *de novo* protein synthesis.

## 1. Introduction

Cell migration and proliferation coupled with controlled cell cycle are beneficial for the repair of sagged and wrinkled skin, dermal, and gastrointestinal wound healing. Cell cycle is a conserved proliferative signaling cascade pathway in mammals and comprises the G_1_, S, G_2_, and M phases. The G_1_/G0 and S transition is a rate-limiting step in the cell cycle and represents the restriction point of the cycle [1]. G_2_/M phase is important for cell multiplication. The basic migratory cycle includes extension of a protrusion edge of a cell, formation of stable attachments near the leading edge of the protrusion, translocation of the cell body forward, and the release of adhesion molecule. All these steps require arrangement of actin cytoskeleton. Small GTPases of the Rho family are key regulators of these cytoskeletal dynamics. Rac-1, Rho-A, and Cdc-42 of Rho family GTPases are required for cell lamellipodial protrusions and activation of wave complex which provides force to cell migration and cell polarity establishment [2]. Like GTPase, cyclin-dependent kinases 1 and 2 are important for cell cycle control [3]. Wound healing requires both migration and proliferation of many cell types like neutrophils, fibroblasts, endothelial cells, and keratinocytes. Fibroblasts play important role in the process of wound healing and maintenance of epidermis dynamics with involvement of Rho-GTPase-dependent activation of basic fibroblast growth factor (bFGF) and collagen. This in turn leads to the activation of Rho-A, thereby facilitating both migration and proliferation of fibroblasts during the process of wound healing [4]. An understanding of the mechanisms that regulate the cell migration and proliferation of dermal fibroblasts cells by a natural compound could be beneficial in devising novel therapies to regulate fibrosis and wound contraction to ultimately improve the wound healing process.

Hyuganatsu, *Citrus tamurana* Hort. ex Tanaka, is one of the predominant citrus crops of Miyazaki, Japan. In recent years, this crop has increased the commercial value especially in food industries. Traditionally the citrus fruit has been used as a supplement to increase digestion and appetite, relieve flatulence and abdominal distension, and help in respiratory difficulties and also in the prevention of cough. Hyuganatsu peel extract (HE) has been reported to inhibit cytochrome P450 3A [5], suppress midazolam 1-hydroxylase activity of human CYP 3A [6] and inhibit hyaluronidase activity [7]. Furthermore, we have tested the efficacy of water soluble extract of Hyuganatsu extract in suppressing bone loss in ovariectomised rats [8]. However, whether it facilitates the process of *in vitro* wound healing and has a beneficial effect on the proliferation and migration of fibroblast cells remains to be explored. Therefore, in the present investigation, we tested the efficacy of HE on human fibroblast cell migration and proliferation and the associated cell cycle pattern and expressions of cell cycle regulatory pathways.

## 2. Materials and Methods

### 2.1. Materials

Lyophilised* Citrus tamurana* peel water extract powder was obtained from Ichimaru Pharcos Co., Ltd. (Gifu, Japan). Alpha medium and FBS were obtained from Sigma Chemicals Co. (St. Louis, MO, USA). Antibiotic cocktail (2500 U/mL penicillin, 2.5 mg/mL streptomycin sulfate, 2.5 mg/mL neomycin) was obtained from Life Technologies Corporation (Invitrogen, Corp., NY, USA). All other chemicals were of pure and molecular grade.

### 2.2. Methods

#### 2.2.1. Cell Culture and Treatment

Human fibroblast cells (TIG-119) were purchased from Health Science Research Resources Bank (HRSBB, Osaka, Japan) and cultured in type-1 collagen coated plates (CELLCOAT, Greiner Bio-One, Germany). Cells were maintained in MEM *α* with glutamine and 5% FBS in 10 cm culture plates. Cells were maintained in antibiotic cocktail at 37°C in a humidified incubator with an atmosphere of 95% air and 5% CO_2_. Cells at passages 2–5 were used for the experiments. All experiments were carried out in FBS deprived MEM +*α* condition. HE was dissolved in sterilized water, sonicated, filtered, and sterilized through 20 *μ*M filter caps (Millex, Millipore Corp., Billerica, MA, USA), and stored at −20°C till further use.

#### 2.2.2. MTT-Based Cytotoxicity and Cell Proliferation Assay

TIG-119 cells were cultured in 96-well culture plates with or without HE (0, 0.1, 0.25, 0.5, 0.75, 1.0, 2.0, and 5.0 mg/mL) for 16 h time duration. The viable cell viability was assessed using MTT assay method [9]. Briefly, 3-[4,5-dimethylthiazol-2-yl]-2,5-diphenyltetrazolium bromide (MTT) was dissolved in phosphate-buffered saline (137 mM NaCl, 2.68 mM KCl, 10 mM Na_2_HPO_4_, 1.76 mM KH_2_PO_4_, and pH 7.4) at a concentration of 5 mg/mL. MTT was added to each well (10 *μ*L per 100 *μ*L medium), and plates were incubated at 37°C for 1 h. The medium was replaced with 100 *μ*L DMSO, and the absorbance for each well was measured at 570 nm on a microplate reader (Biorad, Corp, USA). For cell proliferation assay, cells were cultured in 24-well culture plate with or without different concentration (0, 0.1, 0.25, 0.5, 0.75, and 1.0 mg/mL) of HE. Total viable cell count was done as per MTT method at different time periods of 24, 48, 72, and 96 h.

#### 2.2.3. *In Vitro* Wound Healing Assay

TIG-119 cells were grown in 6-well plates at a density of 3 × 10^6^/mL, and a small linear scratch was created in the confluent monolayer by gently scraping with sterile cell scrapper as per standard methods [10]. Cells were extensively rinsed with medium to remove cellular debris before treating with different concentrations (0, 0.1, 0.25, 0.5, 0.75, and 1.0 mg/mL) of HE in FBS deprived condition. A positive control, prostaglandin I2 (PG12) analogue, and beraprost sodium (Kaken Pharmaceuticals, Co., Fukuoka, Japan) were used separately to judge the rate of cell migration. Twenty-four hours later, images of the migrated cells were taken using digital camera (Nikon, Tokyo, Japan), connected to the inverted microscope (Nikon, TMS-F, Japan), and analyzed by image analysis software (Image J, National Institutes of Health, Bethesda, MD, USA). Extent of wound healing was determined by the distance traversed by cells migrating into the denuded area. Representative data is cumulative of three independent experiments.

#### 2.2.4. Cell Cycle Analysis by Flow Cytometer

Fibroblasts cells (TIG-119) were treated with different concentrations of HE (0, 0.1, 0.25, 0.5, 0.75, and 1.0 mg/mL) for 24 h and fixed in cold 70% ethanol at 4°C. Cells were stored in the fixative at −20°C for 1 h. Following fixation, cells were centrifuged at 800 ×g for 5 min, resuspended in phosphate-citrate (PC) buffer at room temperature for 30 min, and again centrifuged at 1000 ×g for 5 min. The cells were resuspended in 800 *μ*L PBS before incubation in 100 *μ*L each of 100 *μ*g/mL RNase A and 0.01% propidium iodide. Flow cytometry analysis was performed after 30 min using Guava Cell Cycle Assay Mini Flow Cytometer (Millipore, Billerica, MA, USA).

#### 2.2.5. Western Blotting

TIG-119 cells were plated at a density of 3 × 10^6^ cells/mL in 80 mm collagen coated tissue culture dishes (CELLCOAT, Greiner Bio-One, Germany) and incubated with or without HE (0, 0.1, 0.25, 0.5, 0.75, and 1.0 mg/mL) for different time intervals. Cells were washed twice with ice-cold PBS and lysed in a buffer containing 50 mM Tris (pH 7.4), 150 mM NaCl, 1% Triton X-100, 1 mM EDTA, 1 mM EGTA, and 1 mM phenylmethanesulfonyl fluoride. Proteins from lysates were separated on polyacrylamide gels and transferred onto polyvinylidene difluoride (PVDF) membranes. After blocking with blocking solution (EZ-20, Atto Corp, Tokyo, Japan), the membranes were probed with antibodies against Rac-1, Rho-A, Cdc-42 (Santa Cruz, CA, USA), Cdk-1, and Cdk-2 (Cell signaling, Beverly, MA, USA) followed by anti-rabbit peroxidase-conjugated secondary IgG-2 antibodies (Cell signaling, Beverly, MA, USA). Finally, the protein bands were visualized with an enhanced chemiluminescence kit (Amersham, Buckinghamshire, UK). Beta-actin was used as loading control. Relative expression signal intensities were quantified by densitometric analysis. 

#### 2.2.6. Semiquantitative Real-Time PCR

Total RNA was isolated from HE-treated groups using one step RNA isolation kit (ZyGEM Corp., Hamilton, New Zealand), and cDNA was prepared using Transcription High Fidelity cDNA synthesis kit (Roche Diagnostics, Indianapolis, IN, USA). RT-PCR was carried out as per standard procedures using ReverTra Ace enzyme (Toyobo, Tokyo, Japan). 

Primer sequences were as follows:

 Rac-1: forward: 5′-CCCTATCCTATCCGCAAACA-3′,
 reverse: 5′-CGCACCTCAGGATACCACTT-3′;
 Rho-A: forward: 5′-CATCCGGAAGAAACTGGT-3′,
 reverse: 5′-TCCCACAAAGCCAACTC-3′;
 Cdc-42: Forward: 5′-CATCCGGAAGAAACTGGT-3′,
 reverse: 5′-TCCCACAAAGCCAACTC-3′;
 Cdk-1: forward: 5′-GGGTAGAGGAGGTGCGGGC-3′,
 reverse: 5′-GCGATGGCCCAGCTCCTC-3′;
 Cdk-2: forward: 5′-CGCTTCATGGAGAACTTC-3′;
 reverse: 5′-GAAGTTCTCCATGAAGCG-3′.

#### 2.2.7. Translation and Transcription Inhibition Assays

Sub confluent cells were pretreated for 2 h with 10 *μ*g/mL of the translational inhibitor, cycloheximide (CHX) in serum-free medium. The cells were then treated with or without HE (0.75 mg/mL) and incubated further for 6 h. Total RNA was isolated, and expression levels of Cdk-1 and Cdk-2 were measured with *β*-actin as internal control. DRB, a transcriptional inhibitor, was used to inhibit the rate of transcription. Cells were pretreated with 10 *μ*g/mL of DRB for 2 h in serum-free medium to prevent new DNA synthesis and treated with HE (0.75 mg/mL). Zero h represents the time of HE addition. Total RNA was isolated at various time intervals (0, 2, 4, 6, and 8 h), and Cdk-1 and Cdk-2 mRNA expression levels were measured against *β*-actin.

### 2.3. Statistical Analysis

Each experiment was carried out in three independent sets. Mean values and standard deviation were calculated. The Kruskal-Wallis test to compare more than two groups was used to judge the statistical significance. Statistically significant values were set at the level of *P* < 0.05.

## 3. Results and Discussion

In this study, we used human skin fibroblasts (TIG-119) to investigate the effect of HE on the patterns of cellular proliferation, migration, and associated mechanism. The analyzed end points were (i) cell replicative capacity (PDL), (ii) cell cycle changes, (iii)* in vitro* cell migration pattern and (iv) fluctuations of cell cycle target genes and proteins.

### 3.1. Dose-Dependent Activity of HE on Cytotoxicity

**Figure 1:**
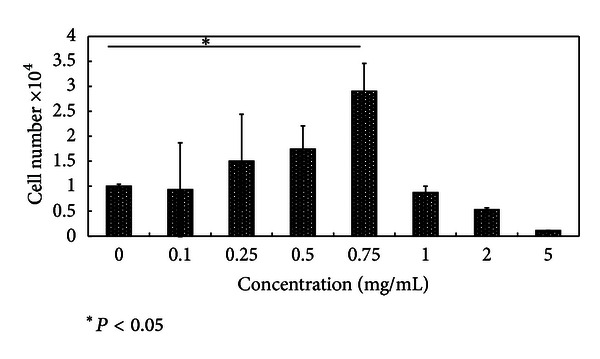
MTT-based cytotoxicity assay. Human skin fibroblast (TIG-199) cells were treated with different concentration of HE (0, 0.1, 0.25, 0.5, 0.75, 1.0, 2.0, and 5.0 mg/mL), and total viable cells were count-based on MTT assay. Each value is the mean ± SD of three independent experiments. Asterisks indicate values which are significantly (*P* < 0.05) different from control.

**Figure 2:**
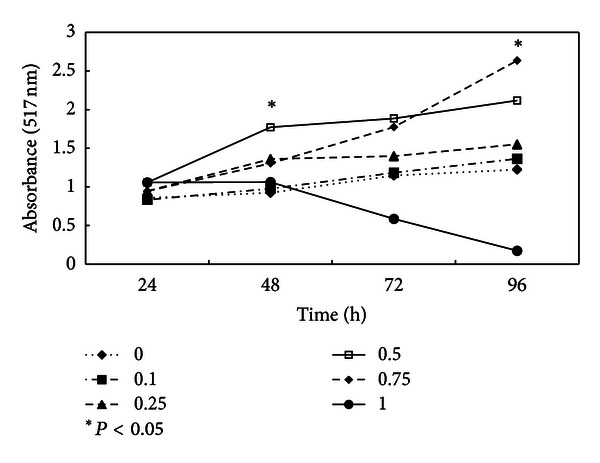
Cell proliferation assay. After the treatment with different (0, 0.1, 0.25, 0.5, 0.75, and 1.0 mg/mL) concentration of HE, increase in total viable cells numbers was counted through MTT dye reduction assay. Cells were counted at different time intervals of 24, 48, 72, and 96 h. Values represent the mean of three independent experiments.

A dose-dependent increase in cell number was noticed during 16 h treatment period with maximum and significant (*P* < 0.05) increase at 0.75 mg/mL concentration level. High concentration of HE (1 mg/mL) inhibited cell proliferation and displayed cytotoxic effect on TIG-119 cells (Figure 1). Lactic dehydrogenase release to the medium during treatment with different concentrations of a compound is the hallmark of cytotoxicity of the compound because of membrane lysis, oxidation reaction of lactate to pyruvate, and subsequent reaction of pyruvate with INT tetrazolium to form formazan. Treatment with 1 mg/mL of HE resulted a 50% decrease in cell number at 72 h. However, at other concentration, there was no difference in cell proliferation between the control groups cells and cells treated with HE (Figure 2). Above results collectively show HE exerts a biphasic action on the fibroblast proliferation. At lower concentrations, HE did not show any cytotoxicity, but at higher concentration of 1 mg/mL, HE displayed cytotoxicity reversed; this could be attributed to the crude nature of the test sample. This study supports several other investigations that reported that natural antioxidants increased the number of oral fibroblast [11] and endothelial cells [12] and modulated the growth of endometrial stromal cells [13].

### 3.2. HE Induces G_2_/M Stage of Cell Cycle

**Figure 3:**
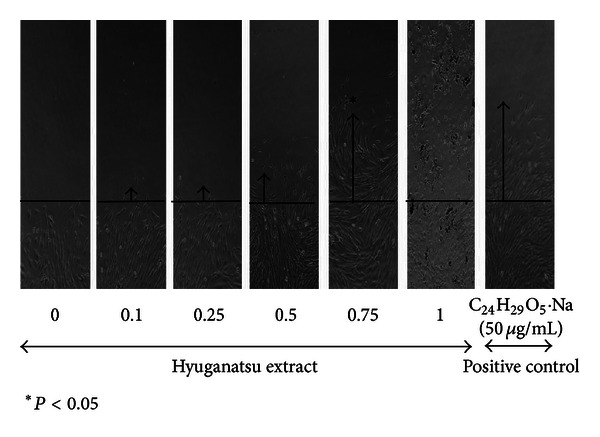
*In vitro* monolayer scratch cell migration assay. Confluent cells were treated with different concentration of HE (0, 0.1, 0.25, 0.5, 0.75, and 1.0 mg/mL) and also beraprost (50 *μ*g/mL) for 16 h time duration. Distance travelled from wound edge to the highest cell point was calculated in mm. Black line indicates the wound edge.

Functional end point of fibroblast cell division and proliferation is cell migration [3] into wounded area [11]. An established *in vitro* scratch assay model was used to quantitatively define human skin fibroblast migration in a monolayer cell model by using NIH Image J software analysis. As depicted in Figure 3, HE dose dependently increased the rate of migration into wounded area up to the concentration of 0.75 mg/mL. The rate of cell migration was similar to that observed in the presence of beraprost, a potent cell migration inducer. Therefore it is proposed that HE is noncytostatic with proproliferative capacity to induce fibroblast cell migration.

**Figure 4:**
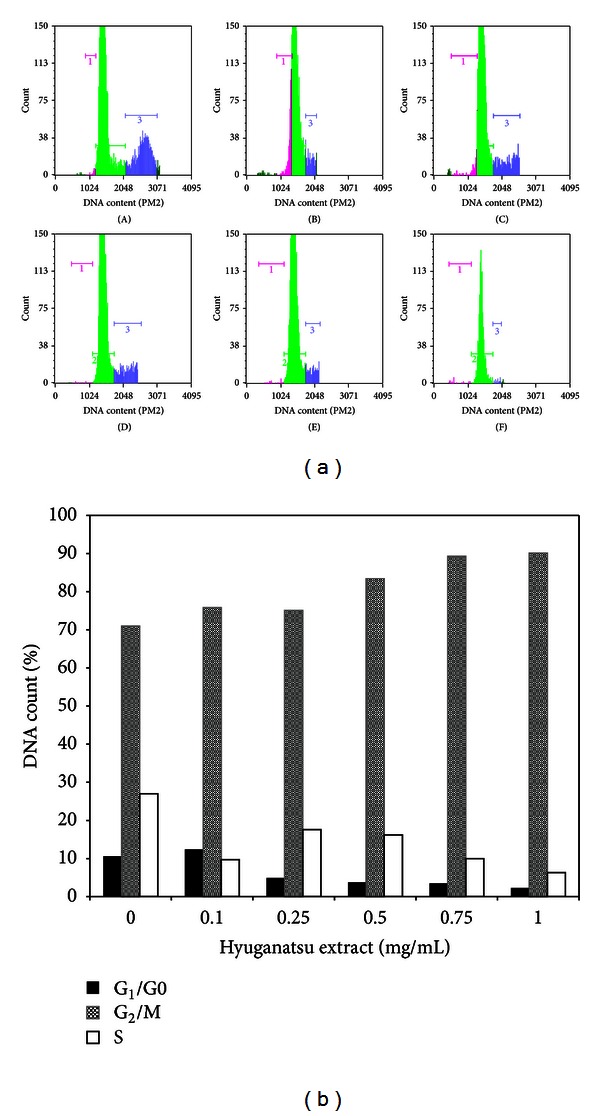
(a) Cell cycle analysis. TIG-119 fibroblast was incubated with different concentrations: (A) 0.0 mg/mL, (B) 0.1 mg/mL, (C) 0.25 mg/mL, (D) 0.5 mg/mL, (E) 0.75 mg/mL, and (F) 1.0 mg/mL of HE for 16. Both floating and adherent cells were collected and analyzed by flow cytometry. The inserts show the proportion of cells in each phase and marked with different colors (pink: G_1_/0 phase: green, G_2_/M phase, and blue: S phase). (b) The data are expressed (% of each phase) as the mean percentage of each phase from independent experiments.

Cell proliferation and migration are hallmarks of cell division. DNA duplication is the key step in cell division and it is controlled by different stages: G_1_/0, G_2_/M, and S phases of cell cycle [14]. Subsequently, we studied if HE treatment affected different stages of cell cycle. As seen in Figure 4, G_2_/M phases of cell cycle were dose dependently increased upon HE treatment with highest value obtained with 0.75 mg/mL treatment. As depicted in Figure 4(b), there were no significant differences in percentage of DNA count at G_2_/M phase (21.2, 9.7, 17.6, 16.2, 10.1, and 16.3) and S phase (10.2, 12.4, 3.9, 2.5, 2.3, and 0.7) between different treatment groups, while DNA content in G_1_/0 phase (50.2, 53.3, 59.7, 82.1, 89.4, and 63.3) varied significantly. This shows that HE does not arrest cells in G_2_/M phase but induces the initiation of DNA synthesis. Several lines of evidence support a molecular mechanism in the response to natural compounds stimulation that does arrest the G_1_/0 phases, and increases G_2_/M phase in fibroblast [15, 16].

### 3.3. HE Acts Differentially on Rho Family GTPase and Cdk

**Figure 5:**
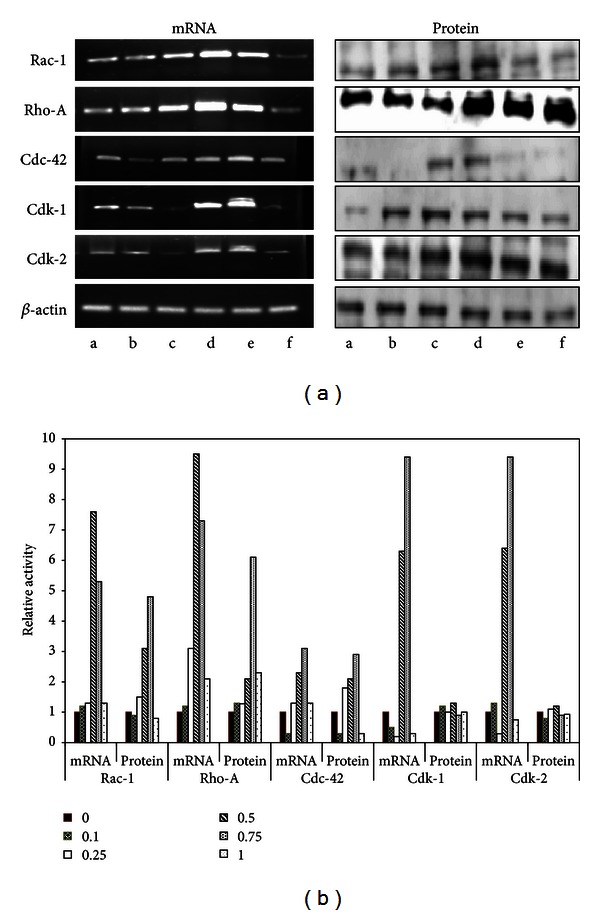
RT-PCR and WB analysis. (a) Western blot analysis and semiquantitative reverse transcription analysis of Rac-1, Rho-A, Cdc-42, Cdk-1, Cdk-2, and *β*-actin. Cells were treated with different concentration of HE (0, 0.1, 0.25, 0.5, 0.75, and 1.0 mg/mL) and processed for RT-PCR and W.B analysis for genes (right panel) and proteins (left panel), respectively. Data here represents the picture from one of three independent experiments. (b) Relative values of mRNA and protein expression which are normalised against *β*-actin.

Fibroblast cell proliferation and migration phenomena are principally governed by Rho family GTPase like Rac-1, Rho-A, and Cdc-42 [4]. The cell cycle phases are coordinated by the expression and/or activation of regulatory proteins, like cyclins (e.g., cyclin A, D, and E), cyclin-dependent kinases (Cdk) mainly Cdk-1 and -2, and Cdk inhibitors. Both cyclins and cyclin-dependent kinases have also been implicated in the formation of actin cytoskeleton in mammalian fibroblast cells [17]. In order to gain an insight into the mechanisms of HE action, we studied the expression of Rac-1, Rho-A, Cdc-42, and Cdk-1 and Cdk-2 mRNA by RT-PCR assay and WB analysis. Cells exposed to 0.1, 0.25, 0.5, and 0.75 mg/mL. HE showed concomitant increase in both mRNA and protein levels of Rac-1, Rho-A, and Cdc-42 (Figure 5(a)). Cyclin-dependent kinases have been identified as key proteins in the G_2_/M transition that help maintain steady-state level of M phase through inhibition of PP 2A/B558 [18]. Molecular mechanisms of mammalian cell migration were first revealed in fibroblasts where Rho-A, Rac-1, and Cdc-42 facilitate the multistep process including the establishment and maintenance of polarity, formation of actin-rich protrusions, remodeling of adhesive contacts, and generation of force. Our results revealed that HE (0.75 mg/mL) induces levels of Rho-A, Rac-1, and Cdc-42 (Figure 5(a)) and helps in cell migrations as shown in Figure 3. Results also showed that mRNA levels of Cdk-1 and -2 increased significantly (Figure 5(a)). However HE did not induce Cdk-1 & -2 protein levels (Figures 5(a) and 5(b)). This observation leads us to hypothesie that HE extract differentially acts on mRNA and protein level working either at transcriptional or translation stage. 

**Figure 6:**
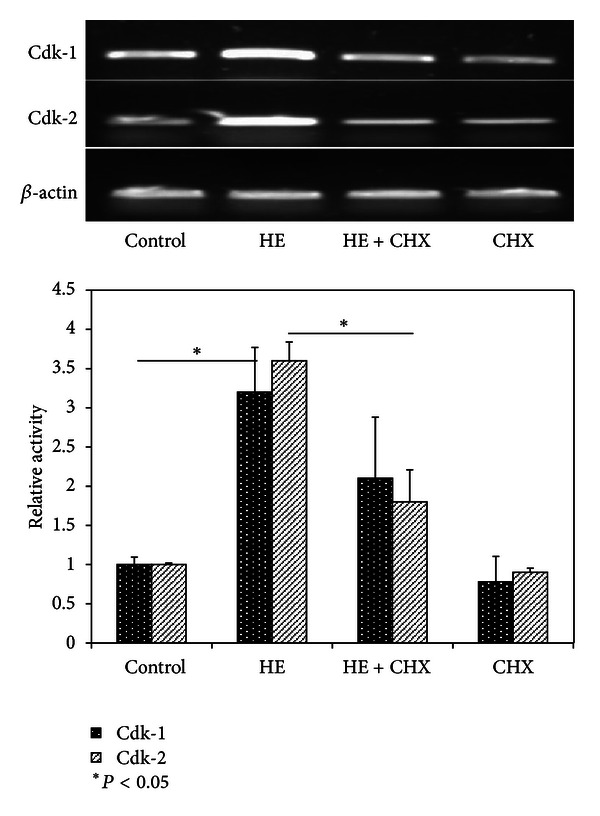
Translation inhibition assay. Effect of cycloheximide (CHX) on HE-induced Cdk-1 and -2 mRNA expression. TIG-119 cells were pretreated for 2 h with 10 *μ*g/mL CHX in serum-free medium and then treated with or without 0.75 mg/mL/mL of HE for 6 h. Total RNA was isolated and analysed for mRNA signals. Relative values of gene expression were depicted. Data represents the values of three independent experiments. Significant values were calculated by Student's *t*-test (*P* < 0.05).

**Figure 7:**
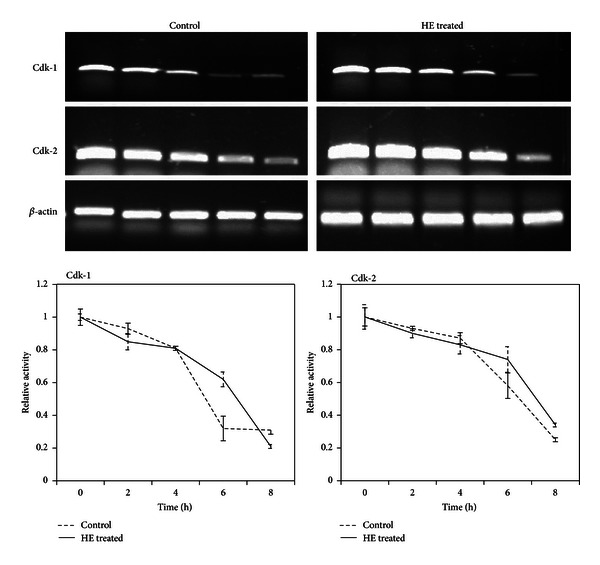
Transcription inhibition assay. Cdk-1 and -2 mRNA expression levels were measured following the treatment of fibroblasts cells with transcription inhibitor, DRB. Cells were pretreated with DRB for 2 h in serum free medium. Time 0 represents the time of HE addition. Total RNA was collected at various times thereafter and analysed by RT-PCR. Relative gene expressions were calculated plotted.

In order to prove the above hypothesis, we first treated subconfluent cells with HE (0.75 mg/mL) with/or without the translational inhibitor, CHX for 6 h, and analysed Cdk-1 and -2 mRNA expressions. As shown in Figure 6, HE alone (0.75 mg/mL) caused about 3-fold increases in both Cdk-1 and -2 mRNA activities; however, CHX abolished the effect of HE. Interestingly, CHX *per se* did not have any effect on Cdk-1 and -2 mRNAs. It is possible that induction of T Cdk-1 and Cdk-2 by HE could depend on some protein factor, that is, inhibited by CHX treatment. We further checked the status of regulation at the transcription level. Subconfluent cells were conditioned for 2 h with DRB to inhibit ongoing transcription prior to treatment with HE (0.75 mg/mL) for varying time period.  Figure 7 reveals the influence of DRB on Cdk-1 & -2 mRNA expression. The mRNA levels in both control as well as HE-treated group diminished with time course. HE failed to overexpress both Cdk-1 and -2 mRNA in the event of transcription inhibition. This implied that ongoing transcription was necessary for the HE action on Cdk-1 and -2 expressions. 

The cyclin-E-dependent kinase Cdk-2 plays a crucial role in cell cycle progression, and the activity of Cdk-2 has been shown to be adhesion dependent and also correlates with mitogenic activity [19]. Taking into consideration the translation and transcription inhibition assays, we conclude that Cdk-1 and -2 regulations by HE are governed at transcription stage where *de novo* protein synthesis is required. 

## 4. Summary

The evidence reported in the current study, combined with the relative safety of HE, suggests that HE has the potential to be developed as a new non-toxic nutraceutical agent for treating skin disorders. The results reported here should stimulate further research into identifying the active molecule which induces the coordinated cell migration and proliferation response in wounded tissues and thereby promotes a better understanding of this fundamental homeostatic process.
